# Preparation and Thermogravimetric and Antimicrobial Investigation of Cd (II) and Sn (II) Adducts of Mercaptopyridine, Amino Triazole Derivatives, and Mercaptothiazoline Organic Ligand Moieties

**DOI:** 10.1155/2021/6638229

**Published:** 2021-04-16

**Authors:** Ahmed Gaber, Walaa F. Alsanie, Robson F. de Farias, Moamen S. Refat

**Affiliations:** ^1^Department of Biology, College of Science, Taif University, P.O. Box 11099, Taif 21944, Saudi Arabia; ^2^Department of Clinical Laboratories Sciences, The Faculty of Applied Medical Sciences, Taif University, P.O. Box 11099, Taif 21944, Saudi Arabia; ^3^Departamento de Química, Universidade Federal de Roraima, Cx. Postal 167, Boa Vista 69301-970, Roraima, Brazil; ^4^Department of Chemistry, Faculty of Science, Taif University, P.O. Box 11099, Taif 21944, Saudi Arabia

## Abstract

The solid adducts of SnCl_2_.(3amt).H_2_O, SnCl_2_.2(3amt).H_2_O, CdCl_2_.(3amt), CdCl_2_.2(3amt), SnCl_2_.(2mct).0.5H_2_O, SnCl_2._2(2mct), CdCl_2_.(2mct), CdCl_2_.2(2mct).H_2_O, SnCl_2_.(2mcp).1.5H_2_O, >_2_.2(2mcp).4H_2_O, CdCl_2_.(2mcp), CdCl_2_.2(2mcp), SnCl_2_.(4amt).4H_2_O, SnCl_2_.2(4amt).1.5H_2_O, CdCl_2_.(4amt).H_2_O, and CdCl_2_.2(4amt) (where the 3amt, 4amt, 2mct, and 2mcp represent 3-amino-1,2,4-triazole, 4-amino-1,2,4-triazole, 2-mercaptothiazoline, and 2-mercaptopyridine simple organic chelates, respectively) were prepared using a solid-state route and investigated by CHN elemental analysis and infrared spectroscopy. Additionally, we investigated the thermogravimetric characterization and antimicrobial proprieties. It is verified that for 3amt and 4amt adducts, the coordination occurs through nitrogen atom. For 2mct compounds, the coordination occurs through nitrogen (Sn) or sulfur (Cd). For 2mcp adducts, both coordination sites nitrogen and sulfur are involved. By examination of TG curves, it is confirmed that for each hydrated compounds, the first mass loss step is linked with the release of water molecules followed by the release of ligand molecules and sublimation of the metal chloride. Furthermore, it is verified that, considering only the release of ligand molecules (3amp, 4amp, 2mct, or 2mcp), the cadmium adducts are always more stable than the correspondent tin adducts probably due to the formation of cross-linking bonds in these compounds. Finally, of these 16 adducts, 14 showed antimicrobial activities against different bacterial and fungal strains.

## 1. Introduction

In the past decades, the problem of multidrug-resistant microorganisms has reached an alarming level worldwide, and the synthesis of new antimicrobial compounds has become an urgent need to treat microbial infections. Organic compounds that include heterocyclic ring systems continue to attract significant interest due to their wide range of biological elements [1]. The nucleus 1,2,4-triazole is incorporated into a variety of important therapeutic agents, which mainly exhibit antimicrobial activities [1, 2]. Among the various five-membered heterocyclic systems, 1,2,4-triazoles and 1,3,4-thiadiazoles and their derivatives gain importance because they constitute the structural features of many bioactive compounds [2]. Triazole and thiadiazole rings are known to be included in the structure of various drugs [3, 4]. From these classes of heterocyclic compounds, the synthesis of novel derivatives of 1,2,4-triazole-3-thionate and 2-amino-1,3,4-thiazole has attracted great interest due to various biological properties such as antibacterial [5, 6], antifungal [7], antituberculosis [8, 9], interferon [10], antioxidant [11], antitumor [12], anti-inflammatory [13, 14], and anticonvulsant [15].

The thermogravimetry analysis technique is employed to identify the acceptability level regarding the coordination nature in between the central metal ions and different kinds of interesting biomolecule chelates, such as amino acids [16], caffeine molecule [17], or chemical materials that have a biological behavior as ethylene- and propylene-urea as well as ethylene-thiourea [18]. Moreover, the thermogravimetric information shows very close relationships with the calorimetric data [19] and the spectral data [20].

**Figure 1:**
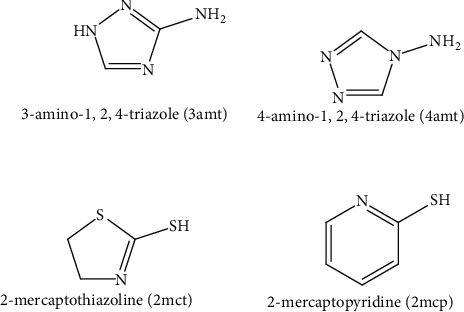
The structural forms of 3amt, 4amt, 2mct, and 2mcp.

The main goal of this article is to investigate the synthesis, thermal analyses, and antimicrobial data of the sixteen solid adducts for the Cd (II) and Sn (II) metal ions coordinated with the 3amt, 4amt, 2mct, or 2mcp organic molecules. The molecular structural formulas of 3amt, 4amt, 2mct, and 2mcp are displayed in Figure 1. The sixteen solid adducts are SnCl_2_.(3amt).H_2_O, SnCl_2_.2(3amt).H_2_O, CdCl_2_.(3amt), CdCl_2_.2(3amt), SnCl_2_.(2mct).0.5H_2_O, SnCl_2._2(2mct), CdCl_2_.(2mct), CdCl_2_.2(2mct).H_2_O, SnCl_2_.(2mcp).1.5H_2_O, SnCl_2_.2(2mcp).4H_2_O, CdCl_2_.(2mcp), CdCl_2_. 2(2mcp), SnCl_2_.(4amt).4H_2_O, SnCl_2_.2(4amt).1.5H_2_O, CdCl_2_.(4amt).H_2_O, and CdCl_2_.2(4amt).

## 2. Materials and Methods

All used reagents were purchased from Sigma-Aldrich and were utilized with no additional purification.

All solid Cd(II) and Sn(II) adducts were prepared by the solid-state pathway by grinding stoichiometric amounts of metal halides and organic moieties (3amt, 4amt, 2mct, and 2mcp) in a mortar for 70 minutes at room temperature (27°C). The prepared solid adducts were dried under vacuum at room temperature for 24 h. This solid-state pathway was successfully used to enhance coordination reactions [21–24] as an alternative to conventional synthesis in solution. The synthesis is performed at room temperature, and where no solvent is used, any unwanted reaction to the metal cation is avoided. The infrared spectra result considering both free organic ligands and sixteen solid adducts proved that there are no free ligand particles after the grinding process.

C, H, and N elemental analysis were performed using a Perkin-Elmer 2400 analyzer. Infrared spectra of the solid adducts as a powder in *situ* KBr discs were scanned using a Gengis II FTIR apparatus within the 4000–400 cm^−1^ range, with a resolution of 4 cm^−1^. Thermogravimetric diagrams under N_2_ atmosphere were analyzed on the Shimadzu TG-50H apparatus with a heating rate of 15°C min^−1^.

Tin(II) and cadmium(II) contents were determined by gravimetry by the direct ignition of the adducts at 600°C for 3 h till constant weight. The residue was then weighted in the forms of SnO and CdO, respectively. The Mohr method uses chromate ions as an indicator in the titration of chloride ions with a silver nitrate standard solution. After all the chloride precipitated as white silver chloride, the first excess of titrant results in the formation of a silver chromate precipitate, which signals the end point.

Preparation of standard AgNO_3_ solution: 9.0 g of AgNO_3_ was weighed out, transferred to a 500 mL volumetric flask, and made up to volume with distilled water. The resulting solution was approximately 0.1 M. This solution was standardized against NaCl. Reagent-grade NaCl was dried overnight and cooled to room temperature. 0.25 g portions of NaCl were weighed into Erlenmeyer flasks and dissolved in about 100 mL of distilled water. In order to adjust the pH of the solutions, small quantities of NaHCO_3_ were added until effervescence ceased. About 2 mL of K_2_CrO_4_ was added, and the solution was titrated to the first permanent appearance of red Ag_2_CrO_4_.

The antimicrobial activity of all adducts were performed as previously explained in detail by Gaber et al. [21]. *Escherichia coli* and *Pseudomonas aeruginosa* were used as Gram-negative bacteria, whereas *Bacillus subtilis* and *Staphylococcus aureus* were used as Gram-positive bacteria. In addition, *Aspergillus flavus* and *Candida albicans* were used as fungal strains. Diameters of the inhibition zones around the hole were calculated [21].

## 3. Results and Discussion

Table 1 shows the data of the elemental analysis. These results are like the proposed formulas. Additionally, the main infrared bands are displayed in Tables 2–5. Before a discussion on the infrared data, it is important to note that considering (3amt and 4amt) and (2mct and 2mcp), organic molecules have a rich electron donor sites through the lone pair of electrons presented on the nitrogen and sulfur atoms, respectively. Moreover, for the four chelates, there is more than one potential coordination site, which makes them able, at the very first moment, to act as cross-links.

In case of 3amt adducts, the overall decrease observed for the symmetrical and asymmetrical N-H bands suggests that the NH_2_ group is engaged with the coordination. Furthermore, positive shifts observed for the *δ*_b_ bands reinforce this statement. It is worth noting that the observed shifts are more intense to Sn(II) adducts than to Cd(II), which is probably due to the higher acidity of Sn(II) (larger nuclear effective charge: 5.65 for Sn and 4.35 for Cd). It is verified that the symmetrical N-H bands are more sensitive to this acidity difference since a positive shift is observed for Cd(II) adducts, whereas a negative shift of this band is verified to Sn(II) adducts.

**Figure 2:**
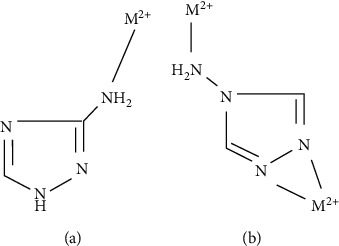
Schematic representation of the suggested coordinative characteristics for (a) 3amt and (b) 4amt adducts.

In case of 4amp approximation, the same general orientation is observed for asymmetric and symmetric N-H bands. This fact indicates that in this case, NH_2_ is involved to a slight degree in the metallic coordination. This hypothesis is reinforced by the fact that for 4amp, the ringed breathing bands exhibit a negative shift (compared to free chelates and synthesized solid adducts), whereas positive shifts are observed in 3amp. Therefore, for 4amp adducts, the two “isolated” nitrogen atoms are the main coordination sites. Suggested coordination modes for 3amp and 4amp molecules are shown schematically in Figure 2.

**Figure 3:**
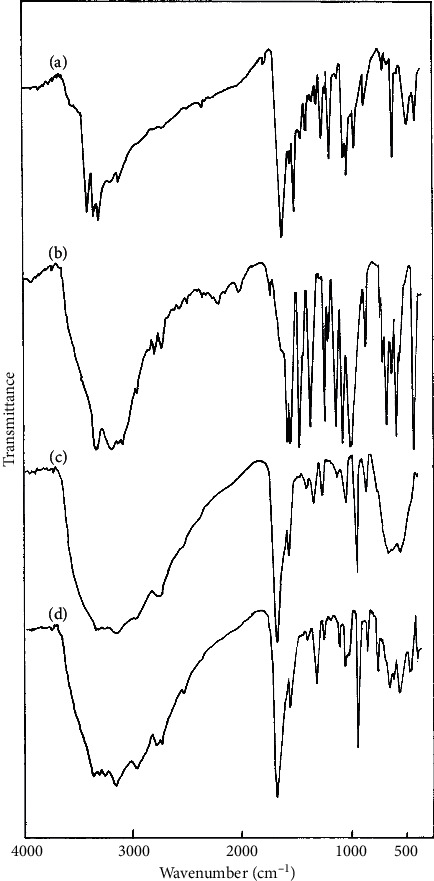
Infrared spectra for (a) CdCl_2_-(3amt), (b) CdCl_2_-2(3amt), (c) SnCl_2_-(3amt).H_2_O, and (d) SnCl_2_-2(3amt).H_2_O.

As explicatory examples, the infrared spectra of 3amt solid adducts are shown in Figure 3.

In case of 2mct adducts, positive shifts of the *ν*C = N band are observed for Sn(II) adducts, whereas negative shifts are verified to Cd(II) adducts. Such fact suggests a coordination through nitrogen to Sn(II) and a coordination through sulfur to Cd(II) in agreement with the fact that the nitrogen atom is a hard base and that Sn(II) is a harder acid than Cd(II).

For 2mcp adducts, the negative shifts observed for the *ν*(C-S); C-SH and *ν*(C=N) aromatic bands suggest that, in this case, both coordination sites N and S are involved in the coordination process for both cations considered.

**Figure 4:**
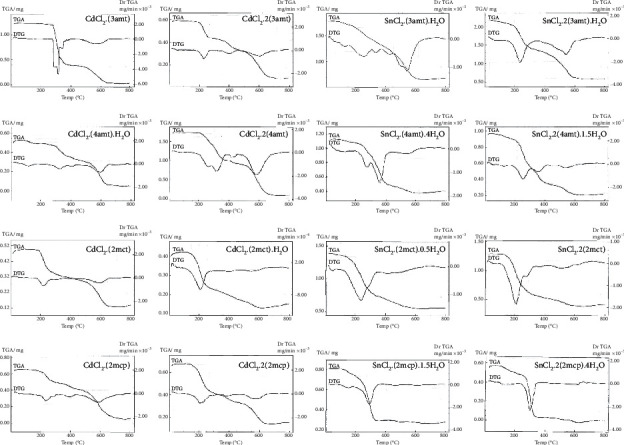
Thermogravimetric curves for the 16 solid adducts.

The data of thermogravimetric curves for the 16 solid adducts are demonstrated in Figure 4. The main TG data are elucidated in Table 6.

For each TG curve, the experimental mass losses (±5%) are similar to the proposed formulas. It is possible to verify that for all hydrated compounds, the first mass loss step is associated with the release of water molecules followed by the release of ligand molecules and sublimation of the metal chloride. Furthermore, it is verified that, considering only the release of ligand molecules (3amp, 4amp, 2mct, or 2mcp), the cadmium adducts are always more stable than the correspondent tin adducts. Since the infrared data suggest that the metal-to-ligand interaction is higher for tin adducts, this last result is an expected one, unless we take into account that the cadmium adducts generally polymerize [22–27] and so there is, probably for these compounds, the formation of cross-linking bonds, leading to more stable compounds, from a thermal point of view.

**Figure 5:**
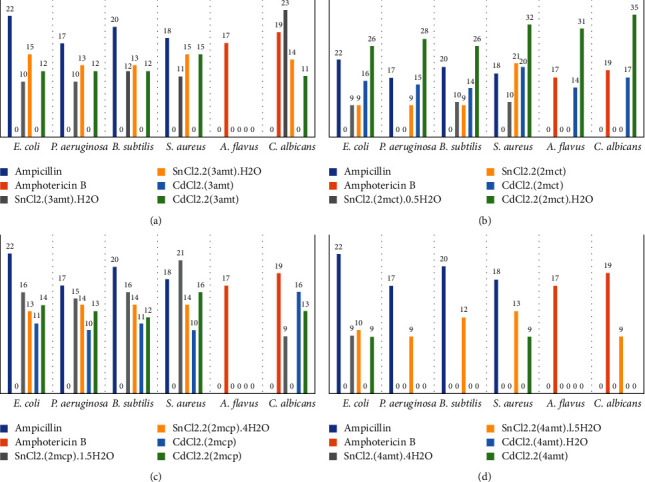
The antimicrobial effects of the 16 solid adducts. The number above the column indicates the inhibition zone diameter.

The antimicrobial effect of the adducts was measured against a variety of microorganisms including bacteria and fungus (Table 7 and Figure 5). The no-growth zones around the hole indicated the inhibiting activity of the adducts on the microbe. These were calculated and compared with the ampicillin as an antibacterial agent or amphotericin B as an antifungal agent. The adduct CdCl_2_.2(2mct).H_2_O and CdCl_2_.(2mct) showed the highest antimicrobial activities followed by SnCl_2_.(2mcp).1.5H_2_O among all other adducts. On other hand, the CdCl_2_.(3amt) and CdCl_2_.(4amt).H_2_O have no effect on any bacteria or fungal strains (Table 7). The antimicrobial activities of these adducts might be caused by a direct interaction of Cd (II) or Sn (II) ions with proteins, enzymes, nucleic acids, and membranes of microbe cells.

## 4. Conclusion

The adducts SnCl_2_.(3amt).H_2_O, SnCl_2_.2(3amt).H_2_O, CdCl_2_.(3amt), CdCl_2_.2(3amt), SnCl_2_.(2mct).0.5H_2_O, SnCl_2._2(2mct), CdCl_2_.(2mct), CdCl_2_.2(2mct).H_2_O, SnCl_2_.(2mcp).1.5H_2_O, SnCl_2_.2(2mcp).4H_2_O, CdCl_2_.(2mcp), CdCl_2_.2(2mcp), SnCl_2_.(4amt).4H_2_O, SnCl_2_.2(4amt).1.5H_2_O, CdCl_2_.(4amt).H_2_O, and CdCl_2_.2(4amt)—where 3amt = 3-amino-1,2,4-triazole; 4amt = 4-amino-1,2,4-triazole; 2mct = 2-mercaptothiazoline; and 2mcp = 2-mercaptopyridine—were synthesized by a solid-state route and characterized by CHN elemental analysis and infrared spectroscopy. A thermogravimetric study was also performed. It is verified that, for all compounds, the monoadducts are the most stable ones. Such fact agrees with a higher ionic and covalent character of the metal-ligand bond for such compounds. From the result, it can be concluded that 14 of the 16 compounds have a good biological activity against these microorganisms.

## Figures and Tables

**Table 1:** Mass weight and carbon, hydrogen, and nitrogen elemental analysis data for the examined compounds.

Adducts	M.wt	% C		% H		% N	
		Calc.	Found	Calc.	Found	Calc.	Found
SnCl_2_.(3amt).H_2_O	291.68	8.22	8.11	2.05	2.02	19.19	18.94
SnCl_2._2(3amt).H_2_O	375.76	12.77	12.25	2.66	2.61	29.81	29.44
CdCl_2._(3amt)	267.37	8.97	8.89	1.49	1.45	20.94	20.73
CdCl_2_.2(3amt)	351.47	13.65	13.62	2.27	2.20	31.87	31.48
SnCl_2_.(2mct).0.5H_2_O	317.81	11.33	10.99	1.89	1.84	4.40	4.35
SnCl_2_.2(2mct)	428.02	16.82	16.32	2.33	2.28	6.54	6.43
CdCl_2_.(2mct)	302.52	11.90	11.74	1.65	1.59	4.62	4.55
CdCl_2_.2(2mct).H_2_O	439.73	16.37	16.22	2.73	2.68	6.37	6.18
SnCl_2_.(2mcp).1.5H_2_O	327.77	18.31	18.19	2.44	2.41	4.27	4.15
SnCl_2_.2(2mcp).4H_2_O	483.94	24.80	24.57	3.72	3.68	5.78	5.74
CdCl_2_.(2mcp)	294.48	20.37	20.22	1.69	1.68	4.75	4.72
CdCl_2_.2(2mcp)	405.65	29.58	29.29	2.46	2.45	6.90	6.84
SnCl_2_.(4amt).4H_2_O	345.68	6.94	6.91	3.47	3.41	16.19	15.98
SnCl_2_.2(4amt).1.5H_2_O	384.76	12.48	12.34	2.86	2.79	29.11	28.96
CdCl_2_.(4amt).H_2_O	285.37	8.40	8.34	2.10	2.07	19.62	19.48
CdCl_2_.2(4amt)	351.47	13.65	13.51	2.27	2.26	31.87	31.83

**Table 2:** Major infrared bands (cm^−1^) for 3amt and its Cd(II) and Sn(II) adducts.

3amt	CdCl_2_-(3amt)	CdCl_2_-2(3amt)	SnCl_2_-(3amt)	SnCl_2_-2(3amt)	Assignments
3398 s	3419 ms	3340 ms	3312 w	3349 vw	*v*_as__(N-H)_; NH_2_
3326 mw	3356 w			3310 vw	
	3316 mw			3258 vw	

3182 mw	3213 w	3212 ms	3155 mw	3155 ms	*v*_s__(N-H)_; NH_2_
	3131 w	3153 vw			

1647 vs	1652 vs	1645 w, sh	1688 vs	1677 vs	*δ*_b_(NH_2_)
1590 s	1573 w	1594 vs	1569 ms	1566 ms	*ν*_(C=N)_
1533 s	1537 s	1560 vs			*ν*_(N=N)_
	1480 w	1479 vs			

1418 s	1429 ms	1374 vs	1405 mw	1415 w	Ring breathing bands
1389 ms	1373 w		1339 ms	1326 s	
	1332 ms				

1275 vs	1283 s	1248 vs	1257 ms	1263 w	*ρ*_r_ (NH_2_)
1217 vs	1250 vw	1213 ms	1125 vw	1125 ms	*ν*_(C-N)_
	1219 vs	1144 vs			
	1144 w				

1045 vs	1088 w	1083 vs	1048 s	1066 s	*ρ*_w_ (NH_2_)
945 vs	1057 s	1011 vs	951 vs	950 vs	
	991 s				

873 vs	901 s	884 s	860 ms	867 s	*ρ*_t_ (NH_2_)
830 s	740 ms	747 sh	748 w, sh	773 ms	
729 vs	693 vw	726 ms			
	644 vs	694 vs			
		642 ms			

s = strong; w = weak; m = medium; sh = shoulder; v = very; br = broad; *ν* = stretching; *δ* = bending.

**Table 3:** Major infrared bands (cm^−1^) for 4amt and its Cd(II) and Sn(II) adducts.

4amt	CdCl_2_-(4amt)	CdCl_2_-2(4amt)	SnCl_2_-(4amt)	SnCl_2_-2(4amt)	Assignments
3312 w	3467 ms	3303 s	3417 w	3418 w, br	*v*_as__(N-H)_; NH_2_
	3368 ms	3258 vw	3317 ms	3277 w, br	
	3307 ms				

3197 w	3199 s	3198 s	3127 ms	3129 w, br	*v*_s__(N-H)_; NH_2_
3139 w	3136 s	3105 s			

1647 vs	1618 vs	1618 vs	1623 vs	1685 w	*δ*_b_(NH_2_)
1533 s	1543 s	1537 s	1539 ms	1631 vs	
				1529 s	

1475 ms	1474 mw	1470 w	1465 vw	1465 vw	Ring breathing bands
1404 s	1398 s	1394 w	1402 vw	1412 vw	
	1341 s	1346 w	1366 vw	1363 vw	
			1318 vw	1323 vw	

1188 s	1209 s	1209 ms	1207 ms	1205 s	*ρ*_r_ (NH_2_)
1074 s	1145 vw	1078 s	1164 vw	1075s	*ν*_(C_–_N)_
	1082 vs		1135 vw		
			1075 s		

1016 vw	1025 s	1015 s	1034 s	1033 s	*ρ*_w_ (NH_2_)
959 ms	980 vs	984 ms	934 ms	935 ms	

873 s	908 w	894 ms	871 ms	875 ms	*ρ*_t_ (NH_2_)
672 w	874 s	845 vw	690 w, sh	661 ms	
	689 ms	686 ms			

s = strong; w = weak; m = medium; sh = shoulder; v = very; br = broad; *ν* = stretching; *δ* = bending.

**Table 4:** Major infrared bands (cm^−1^) for 2mct and its Cd(II) and Sn(II) adducts.

2mct	CdCl_2_-(2mct)	CdCl_2_-2(2mct)	SnCl_2_-(2mct)	SnCl_2_-2(2mct)	Assignments
2852 w	3258 s	3258 s	3443 s, br	3447 ms, br	*v*_s__(C-H)_; –CH_2_
	2948 vw	3136 ms	3206 ms	3207 w	*v*_(O-H)_; H_2_O
		2998 w	3144 vw	3136 w	
		2947 w	2997 w	2997 w	
		2844 w	2929 w	2848 vw	
			2845 w		

2709 mw	—	—	—	—	*ν*_(SH)_
2565 mw					

1518 vs	1515 vs	1515 vs	1539 vs	1539 w, sh	*ν*_(C=N)_
				1514 s	Ring breathing bands

1260 w	1308 s	1305 s	1307 s	1294 s	*v*_as__(C-N)_
1217 w	1250 vw	1249 w	1253 w	1250 vw	*v*_s__(C-N) + _ *ν*_(C-C)_
1160 w	1192 ms	1193 s	1208 w	1205 vw	*ν*_(C-S); C – SH_
1102 s	1142 w	1045 vs	1167 w	1041 s	
	1045 vs		1038 s		

s = strong; w = weak; m = medium; sh = shoulder; v = very; br = broad; *ν* = stretching; *δ* = bending.

**Table 5:** Major infrared bands (cm^−1^) for 2mcp and its Cd(II) and Sn(II) adducts.

2mcp	CdCl_2_-(2mcp)	CdCl_2_-2(2mcp)	SnCl_2_-(2mcp)	SnCl_2_-2(2mcp)	Assignments
—	3458 ms, br	3448 ms, br	3421 ms, br	3423 ms, br	*ν*_(OH)_; H_2_O
	3196 ms	3172 ms	3216 ms	3073 vw	
	3126 ms		3135 w		
	3087 s				

2709 mw	—	—	—	—	*ν*_(SH)_
2537 mw					

1576 vs	1602 vs	1585 vs	1582 vs	1578 vs	Ring breathing bands
1504 s	1517 s	1513 s	1550 sh	1551 sh	
1446 ms	1443 s	1443 s	1517 s	1438 vs	
1418 s	1378 s	1370 s	1438 vs	1366 w	
			1360 s		

1275 ms	1262 s	1252 s	1259 vs	1262 s	*ν*_(C=N)_; aromatic
1246 ms	1160 ms	1163 s	1155 w, sh	1179 vw	*δ*(C-H); in-plane bend
1188 vs					

1145 vs	1134 vs	1132 vs	1131 s	1136 s	*ν*_(C-S); C – SH_
1102 vw	1111 vw	1109 vw	1080 ms	1081 ms	

s = strong; w = weak; m = medium; sh = shoulder; v = very; br = broad; *ν* = stretching; *δ* = bending.

**Table 6:** TG data summary for Sn(II) and Cd(II) adducts with 3amt, 4amt, 2mcp, and 2mcp.

Adduct	Step	*t*_i_ (°C)	Degradation *t*_f_ (°C)	Process onset (°C)	Mass loss (%)
SnCl_2_.(3amt).H_2_O	1	55	382	238	26.7
	2	385	560	473	35.5
SnCl_2._2(3amt).H_2_O	1	45	102	76	5.1
	2	160	445	320	40.0
	3	447	606	517	27.2
CdCl_2._(3amt)	1	280	331	307	53.7
	2	332	406	348	12.8
	3	478	660	569	29.3
CdCl_2_.2(3amt)	1	188	271	234	24.1
	2	348	448	401	16.8
	3	449	681	587	43.0
SnCl_2_.(2mct).0.5H_2_O	1	31.4	83	50	3.3
	2	127	344	237	43.2
	3	346	461	404	7.5
	4	462	615	513	6.2
SnCl_2_.2(2mct)	1	120	260	204	44.0
	2	261	341	288	10.2
	3	342	430	378	4.7
	4	431	595	513	8.9
	5	596	656	607	1.1
CdCl_2_.(2mct)	1	181	276	223	30.2
	2	497	650	583	41.3
CdCl_2_.2(2mct).H_2_O	1	136	276	207	43.2
	2	277	485	384	15.4
	3	486	628	551	9.3
SnCl_2_.(2mcp).1.5H_2_O	1	110	199	154	8.0
	2	200	355	285	55.4
	3	421	654	534	3.1
SnCl_2_.2(2mcp).4H_2_O	1	103	221	154	14.3
	2	222	349	302	82.1
	3	584	670	585	1.8
CdCl_2_.(2mcp)	1	186	309	244	23.3
	2	310	459	391	12.6
	3	460	647	576	47.0
	4	648	751	675	8.6
CdCl_2_.2(2mcp)	1	139	274	215	35.8
	2	276	349	301	5.2
	3	351	482	399	10.1
	4	483	649	572	26.9
SnCl_2_.(4amt).4H_2_O	1	46	145	110	2.8
	2	210	299	269	15.7
	3	300	425	355	37.0
	4	428	616	515	10.2
SnCl_2_.2(4amt).1.5H_2_O	1	63	191	133	6.8
	2	192	319	257	39.2
	3	320	407	358	18.2
	4	497	619	545	14.2
CdCl_2_.(4amt).H_2_O	1	88	176	123	6.7
	2	274	381	328	21.1
	3	382	459	407	7.9
	4	460	655	583	51.3
CdCl_2_.2(4amt)	1	195	289	256	17.9
	2	290	390	324	23.7
	3	392	456	420	4.8
	4	457	679	583	47.5

*t*_i_ and *t*_f_ are the initial and final temperatures of the thermal degradation process, respectively.

**Table 7:** Antimicrobial activities (inhibition zone diameter, mm/µg sample) of papaverine and its metal complexes against Gram-positive bacteria, Gram-negative bacteria, and two types of fungi.

The adducts	Gram-negative bacteria		Gram-positive bacteria		Fungi	
	*E. coli*	*P. aeruginosa*	*B. subtilis*	*S. aureus*	*A. flavus*	*C. albicans*
Control: DMSO	0.0	0.0	0.0	0.0	0.0	0.0
Ampicillin (Antibacterial agent)	22	17	20	18	0.0	0.0
Amphotericin B (Antifungal agent)	0.0	0.0	0.0	0.0	17	19
SnCl_2_.(3amt).H_2_O	10	10	12	11	0.0	23
SnCl_2_.2(3amt).H_2_O	15	13	13	15	0.0	14
CdCl_2_.(3amt)	0.0	0.0	0.0	0.0	0.0	0.0
CdCl_2_.2(3amt)	12	12	12	15	0.0	11
SnCl_2_.(2mct).0.5H_2_O	9	0.0	10	10	0.0	0.0
SnCl_2._2(2mct)	9	9	9	21	0.0	0.0
CdCl_2_.(2mct)	16	15	14	20	14	17
CdCl_2_.2(2mct).H_2_O	26	28	26	32	31	35
SnCl_2_.(2mcp).1.5H_2_O	16	15	16	21	0.0	9
SnCl_2_.2(2mcp).4H_2_O	13	14	14	14	0.0	0.0
CdCl_2_.(2mcp)	11	10	11	10	0.0	16
CdCl_2_.2(2mcp)	14	13	12	16	0.0	13
SnCl_2_.(4amt).4H_2_O	9	0.0	0.0	0.0	0.0	0.0
SnCl_2_.2(4amt).1.5H_2_O	10	9	12	13	0.0	9
CdCl_2_.(4amt).H_2_O	0.0	0.0	0.0	0.0	0.0	0.0
CdCl_2_.2(4amt)	9	0.0	0.0	9	0.0	0.0

## Data Availability

The data used to support the findings of this study are included within the article.
